# Innovative Targeting Strategies in Drug Therapy for Inflammatory Diseases: Mechanistic Approaches

**DOI:** 10.1155/2015/891676

**Published:** 2015-03-30

**Authors:** Duen-Suey Chou, Joen-Rong Sheu, Philip A. Thomas, Muniyan Sakthivel

**Affiliations:** ^1^Graduate Institute of Medical Sciences, College of Medicine, Taipei Medical University, Taipei 110, Taiwan; ^2^Department of Microbiology, Institute of Ophthalmology, Joseph Eye Hospital, Tiruchirappalli, Tamil Nadu 620 001, India; ^3^Department of Biochemistry and Molecular Biology, University of Nebraska Medical Center, Omaha, NE 68198, USA

The treatment of inflammatory disease is conquered by the administration of anti-inflammatory and immunosuppressive drugs, which defeat the inflammatory burden and recover the disease-related symptoms. Conventional treatment strategies are characterized by limited therapeutic efficacy and the occurrence of adverse drug reactions. Therefore, the progress of novel disease-targeted drug delivery stratagems is proposed for a more effective therapy. Though numerous harmonizing and conventional treatment strategies have been assimilated into experimental and clinical practices, the pertinent molecular mechanisms are still under investigation. Hence, we invited researchers to contribute original research articles as well as review articles to offer solid suggestion to sustain the application of innovative drugs in prevention and treatment of inflammatory diseases.

A paper in this special issue investigates the effect of* Antrodia camphorata,* a rare Taiwanese medicinal mushroom that is popularly known as “niu cheng zhi” in Taiwan, on inflammatory arterial thrombosis-mediated platelet activation and also the pivotal role of protein kinase C in this situation was investigated. This paper demonstrated clearly that* Antrodia camphorata* holds antiplatelet activity via inhibiting Ca^2+^, PKC cascades, and Akt signaling pathway. These alterations may reduce platelet activity and ultimately inhibit platelet aggregation. Another interesting paper in this special issue addresses the same aspect that combinative therapy of* Antrodia camphorata* with aspirin offers enhanced neuroprotective efficacy without increasing side effects. This paper thoroughly planned and demonstrated that* Antrodia camphorata* alone or combined with aspirin enhanced neuroprotective efficacy by reducing brain infarct volume, neurobehavioral score, cerebral blood perfusion, and subarachnoid and intracerebral hemorrhage incidence. This paper also showed that* Antrodia camphorata* alone or with aspirin did not alter the level of hemoglobin, as this treatment is safe and does not cause hemorrhagic incident. The findings of these papers suggested that* Antrodia camphorata* may be a potential therapeutic agent for preventing or treating thromboembolic disorders without causing any major side effects.

This special issue also published an inspiring paper that has investigated the mechanisms of the inhibitory effects of andrographolide, a most active and critical constituent isolated from the leaves of* Andrographis paniculata* in vascular smooth muscle cells (VSMCs) exposed to a proinflammatory stimulus, tumor necrosis factor-*α* (TNF-*α*). This mechanistic study proposed that andrographolide can benefit the treatment of vascular inflammatory diseases, and andrographolide-mediated inhibition of NF-*κ*B activity in TNF-*α*-stimulated VSMCs may occur through the JNK-Akt-p65 signaling cascade, an I*κ*B*α*-independent mechanism. In addition, an interesting piece of work in this special issue examined the effect of an ethanolic extract of* Sanguis draconis*, a kind of dragon's blood resin that was obtained from* Daemonorops draco* (Palmae), on human umbilical vein endothelial cells (HUVEC) under high-glucose stimulation. This paper showed that* Sanguis draconis* attenuated high-glucose induced cell toxicity, nitrite, lipid peroxidation, and reactive oxygen species (ROS) formation in HUVEC. This treatment method abolished phosphorylation of ERK 1/2, NF-*κ*B, VCAM-1, and E-selectin, and it seems to block the breakdown of PARP-116 kDa protein. Furthermore, this paper found that* Sanguis draconis* increased the expression of Bcl-2 and decreased Bax protein expression. The results of this paper suggest that* Sanguis draconis* may have a therapeutic potential in vascular inflammation due to the decreased levels of oxidative stress, apoptosis, and PARP-1 activation. Overall, we expect that this special issue grants progressive awareness to upsurge the therapeutic significance for drug development to treat or prevent inflammatory diseases.

*Duen-Suey Chou* *Duen-Suey Chou* *Joen-Rong Sheu* *Joen-Rong Sheu* *Philip A. Thomas* *Philip A. Thomas* *Muniyan Sakthivel* *Muniyan Sakthivel*

